# The effect of medical cannabis on cognitive functions: a systematic review

**DOI:** 10.1186/s13643-022-02073-5

**Published:** 2022-10-03

**Authors:** Anders Wieghorst, Kirsten Kaya Roessler, Oliver Hendricks, Tonny Elmose Andersen

**Affiliations:** 1 Department of Psychology, University of Southern Denmark, Campusvej 55, 5230 Odense M, Denmark; 2 Danish Hospital for Rheumatic Diseases, University Hospital of Southern Denmark, Sonderborg, Denmark; 3 Department of Regional Health Research, University of Southern Denmark, Odense, Denmark

## Abstract

**Background:**

Cannabis-based medicines are widely used in the treatment of a number of medical conditions. Unfortunately, cognitive disturbances are often reported as adverse events, although conversely, cognitive improvements have been reported. Hence, the objective of the present study was to identify, critically appraise and synthesise research findings on the potential impact of cannabis-based medicines on cognitive functioning.

**Methods:**

Four databases (EMBASE, PsycINFO, PubMed and Scopus) were systematically searched. Studies were included if they provided findings on the impact of cannabis-based medicines in controlled settings on cognitive functioning measured by recognised cognitive tests in human adults. Study participants were required to be their own case-control, and neither studies on abuse, abstinences, patients with severe neurodegenerative diseases nor cancer-related pain conditions were included. Screening, risk of bias assessment and data extraction were conducted independently by two researchers. Findings were tabulated and synthesised by outcome.

**Findings:**

Twenty-three studies were included, comprising a total of *N* = 917. Eight studies used Sativex as the cannabis-based medicine two used Epidiolex, two other studies used sprays, three studies used gelatine capsules, five smoked cannabis, two other and finally one studied cannabis withdrawal. Fifteen studies reported non-significant findings; six reported cognitive impairments; one study found cognitive improvement and a single study found improvement following withdrawal. Thirteen studies had cognitive or neuropsychological functioning as the primary outcome.

**Conclusions:**

Due to a large heterogeneity and methodological limitations across studies, it is not possible to make any definite conclusions about the impact of cannabis-based medicines on cognitive functioning. However, the majority of high-quality evidence points in the direction that the negative impact of cannabis-based medicines on cognitive functioning is minor, provided that the doses of THC are low to moderate. On the other hand, long-term use of cannabis based medicines may still adversely affect cognitive functioning. In the studies that found impaired cognitive functioning to be significant, all of the test scores were either within the normal range or below what would be characterised as a neuropsychologically cognitive impairment.

## Introduction

In 1996, California became the first US state to legalise marijuana for medicinal purposes [1]. Since then, the use of cannabis-based medicines (CBMs) has dramatically increased [2]. CBMs are widely used in the treatment of several conditions and symptoms such as chronic pain, multiple sclerosis, epilepsy, nausea, vomiting and spasticity, to mention but a few [3, 4]. Moreover, CBMs cover a wide number of substances ranging from recreational cannabis used in medical settings with a licence to plant-based cannabidiol (CBD); tetrahydrocannabinol (THC) and combinations thereof (CBD/THC), all prescribed in different doses and combinations. This significant public and scientific interest in CBMs is contrasted by a lack of high-quality studies assessing—not only the effects, but also the potential adverse effects of CBMs [5].

A systematic review including 46 randomised controlled trials with non-cancer pain, Stockings et al. [6] concluded that CBMs were more likely to produce a 30% reduction in pain compared to placebo (*n* = 1734, OR 1.46, 95% CI 1.16–1.84). However, the number needed to treat to achieve this effect was 24, while the number needed to harm was only 6. Importantly, the most common reported adverse event was ‘cognitive or attention disturbances’ with an odds ratio of 5.67 (95% CI 2.72–11.79) compared to placebo. While the findings by Stockings et al. [6] rely on self-reported cognitive disturbances a recent systematic review by Landrigan et al. [7] has assessed the effects of cannabis on cognition in people with multiple sclerosis by synthesising studies using a valid objective measure of cognitive functioning. Landrigan et al. [7] concludes that studies of oral cannabis-based medicinal preparations such as Sativex in general do not affect cognitive functioning compared to controls/placebo treatments. However, studies of whole-plant cannabis were more often associated with negative effects on cognitive functions. Also, it is not known whether long-term or chronic use of oral cannabis-based medical preparations may still negatively affect cognitive functioning.

While most studies agree that acute cannabis intoxication adversely affects cognitive functioning, the impact of CBMs on cognitive functioning is still debated [8]. Additionally, other studies have reported *improved* cognitive functioning as an effect of CBMs [9]. Many studies find CBMs like CBD to have minimal if any negative impact on cognitive functioning. Moreover, the long-term negative effects of CBMs have been questioned, with some studies finding the negative effects reversible after a period of drug abstinence. For instance, Feinstein et al. [10] found that in a group of long-term users with multiple sclerosis (> 5 years), a 28-day-period of abstinence resulted in significant improvements in memory, processing speed and executive function. These mixed findings may be the results of different explanatory and confounding factors, such as conditions that CBMs are used for, product and dose used, prior experience and length of treatment as well as measurement methods and time of testing. Hence, more systematic knowledge about the potential impact of CBMs on cognitive functions is needed.

A more in-depth understanding of the reported adverse effects on cognitive functioning is important for a number of reasons. First, it has not been systematically assessed in different patient groups how different CBMs affect cognition measured by recognised neuropsychological tests. As reported in Stockings et al. [6], the list of recorded adverse effects on cognitions is large, but so are the confidence intervals, indicating that significantly different adverse effects may be experienced depending on the condition and type of CBMs used. It should be noted that the negative impact on cognition was based on self-reports and not recognised neuropsychological tests. Only, Landrigan et al. [7] has assessed the impact on cognitive functioning by synthesising studies using valid cognitive tests, however only in patients with MS.

Also, the impact of CBMs on cognitive functioning is affected by the time of testing. While some studies test patients after a very short treatment period or when the patients may be acutely intoxicated [11–13], other studies test patients after a period of titration [14–17]. Another important factor that may affect cognition is whether patients have prior experiences with medical cannabis or are naive users. Moreover, an almost unlimited number of cognitive tests exist to assess cognitive functioning, which is also reflected in the different tests used across studies. For these reasons, a systematic review is needed to provide an overview.

Hence, the aim of the current review is to uncover the field for potential impact of CBMs on cognitive functioning when used in a controlled setting as part of the treatment for chronic pain or other medical conditions with the exclusion of severe neurodegenerative diseases and cancer-related pain conditions.

## Methods

### Protocol and registration

This review was conducted in accordance with Preferred Reporting Items for Systematic Reviews and Meta-Analyses guidelines (PRISMA) [18], and the review protocol was registered beforehand in PROSPERO (CRD42020127488).

### Eligibility criteria

The rational for this review was to examine the effect of medicinal cannabis on cognitive functioning in all patient populations where CBMs is prescribed as part of the treatment, with the exception of severe neurodegenerative diseases and cancer-related pain conditions. Neurodegenerative diseases were excluded since it would be impossible to determine whether a possible decline in cognitive function would stem from degeneration or CBM. Cancer-related pain conditions were excluded to discriminate between non-cancer and cancer pain, as is the tradition in pain research. Although this discrimination is debated, most of the research literature still use this distinction, why we also choose to do so (see “Limitations of this review” section). Studies with medical cannabis in all its forms were included, provided the study was conducted in a controlled setting with essential details about strength and dose. Consequently, studies such as [19, 20] were excluded, since the cannabis delivery was administered by the patients themselves without any information on strength or the ratio between THC and CBD. Participants had to be their own case-controls with baseline cognitive testing before treatment, followed up by at least one testing while under CBM treatment. Cognitive tests must be recognised or shown to be valid and reliable. Peer-reviewed journal manuscripts were included if they were (a) published between 1996 and 2021 (from legalisation in California till the present time); and (b) involved adult human participants without degenerative brain diseases. No limitations regarding language or geography were applied.

### Inclusion and exclusion criteria

The following inclusion and exclusion criteria were developed prior to screening:

#### Inclusion

1. Medical cannabis in a controlled setting with information about strength and dose of CBM, and with cognitive testing at baseline before treatment and with at least one follow-up testing.
2. Human studies only.
3. Own case-control (baseline, repeated measures design, longitudinal study, cross-over design).
4. Baseline test while *not* under treatment and/or the influence of cannabis or other psychoactive drugs.
5. Re-test conducted while under cannabis treatment and/or the influence.
6. Measures from at least one recognised cognitive test.

#### Exclusion

1. Studies on abuse and/or abstinences.
2. Populations with severe neurodegenerative brain diseases and cancer-related pain conditions.
3. Severe psychiatric diseases, such as schizophrenia or psychosis.
4. Under the age of 18.

### Search strategy

The databases of EMBASE, PsycINFO, PubMed and Scopus were searched for eligible studies. Original searches were conducted from 22 November 2018 through 15 January 2019. The first supplemental search was conducted on 25 February 2020 and the second supplemental search on 15 April 2021.

A separate search strategy for each database was developed. Several initial scoping searches were conducted, from which a few key search terms were extracted. A combination of cognitive domain names and key phrases based on our own knowledge was the starting point for search term harvesting in each database’s thesaurus (if present). Due to an extensive number of terms, including variations of cognitive function, the search was limited to include terms for which there is a broadly recognised tradition and test. Hence, terms such as ‘concentration’ or ‘decision-making’ do not always refer to cognitive functions; rather, the concentration of a substance in a solution or how to make the best decision in treatment. Below is an example of the final search strategy for the PubMed database.

#### PubMed

(“Cannabis”[Mesh] OR “Cannabinoids”[Mesh] OR “Cannabinol”[Mesh] OR “cannabielsoin” [Supplementary Concept] OR “Marijuana Abuse”[Mesh] OR “Marijuana Smoking”[Mesh] OR “Medical Marijuana”[Mesh] OR “Marijuana Use”[Mesh] OR “delta-9-tetrahydrocannabinol dichloroethyl carbamoyl ester” [Supplementary Concept] OR “11-nor-delta(9)-tetrahydrocannabinol-9-carboxylic acid” [Supplementary Concept] OR “Dronabinol”[Mesh] OR “Hashish oil” [Supplementary Concept] OR “nantradol” [Supplementary Concept] OR “desacetylnantradol” [Supplementary Concept] OR “nabiximols” [Supplementary Concept] OR “nabilone” [Supplementary Concept] OR “rimonabant” [Supplementary Concept] OR “canabi*” [All Fields] OR “mari?uana” [All Fields] OR “tetrahydrocannabinol” [All Fields] OR “Dronabinol” [All Fields] OR “Hashish” [All Fields] OR “nabiximols” [All Fields] OR “nabilone” [All Fields] OR “rimonabant” [All Fields])

AND

(“Attention”[Mesh] OR “Cognitive Dysfunction”[Mesh] OR “Decision Making”[Mesh] OR “Distracted Driving”[Mesh] OR “Intelligence Tests”[Mesh] OR “Intelligence”[Mesh] OR “Learning”[Mesh] OR “Memory and Learning Tests”[Mesh] OR “Memory Consolidation”[Mesh] OR “Memory, Episodic”[Mesh] OR “Memory, Long-Term”[Mesh] OR “Memory, Short-Term”[Mesh] OR “Memory”[Mesh] OR “Mental Processes”[Mesh] OR “Multitasking Behavior”[Mesh] OR “Neuropsychological Tests”[Mesh] OR “Psychomotor Performance”[Mesh] OR “Spatial Memory”[Mesh] OR Attention” [All Fields] OR “Cogni*” [All Fields] OR “Decision Making” [All Fields] OR “Distracted Driving” [All Fields] OR “Intelligence Tests” [All Fields] OR “Intelligence” [All Fields] OR “Learning” [All Fields] OR “Memory*” [All Fields] OR “Mental Processes” [All Fields] OR “Multitasking Behavior” [All Fields] OR “Neuropsychological Tests” [All Fields] OR “Psychomotor Performance” [All Fields] OR “Spatial Memory” [All Fields] )

AND (1996:2020[pdat]) AND (journalarticle[Filter])

Word in brackets describe whether the term is a search term or a Medical Subject Heading (MeSH). All search words were checked for being registered as MeSH terms. If this was the case, a search was conducted to examine if it was necessary to include the search term booth as a mesh term with explosion and a search term, as is recommended. This was done to minimise the complexity of the search string, while still yielding the same search results, as some databases had trouble handling the long search string. Similar exercises were done in all databases. Also, reference lists of the final 23 eligible studies were searched for additional references to ensure exhaustiveness.

### Data screening

All references were imported into EndNote X9, where duplicates were identified and removed. The remaining records were exported to the online screening tool www.covidence.org, where the authors AW and TEA independently screened titles and abstracts according to the eligibility criteria. Conflicts were solved in discussion between the screeners.

All references identified as potentially relevant were obtained in full and transferred to full-text screening. Full-text screening was conducted by AW and TEA and conflicts were resolved in discussion.

### Data charting process

A data-charting form was jointly developed by the two reviewers to determine which variables to extract. The two reviewers independently extracted data and discussed the results in case of disagreement. Extracted data were: article characteristics (author, year, country and settings), population characteristics (patient group, e.g. MS, Tourette’s syndrome), sample size (*n*), mean age and sex), study characteristics (design, tests, primary and secondary outcome), cannabis treatment characteristics (period, method of delivery, content CBD and/or THC), placebo content and finally the main findings concerning cognitive functions.

### Quality assessment

Risk of bias was assessed using The Effective Public Health Practice Project (EPHPP) [21] tool, which was developed to assess the methodological quality of primary studies with a variety of study designs [21]. It consists of six component ratings: (1) selection bias, (2) study design, (3) confounders, (4) blinding, (5) data collection method and (6) withdrawals and dropouts. Each component is rated as either weak, moderate or strong. A *strong* global rating is achieved if there are no weak ratings; *moderate* if there is one weak rating and *weak* if there are two or more weak ratings. The tool was slightly modified, since the component domain (c) “confounders” was primarily relevant for randomised controlled studies, where control for group differences is important. In the present study, the populations are their own controls, making this component less relevant. Again, the two reviewers independently assessed the studies and discussed the results in case of disagreement.

## Results

**Fig. 1:**
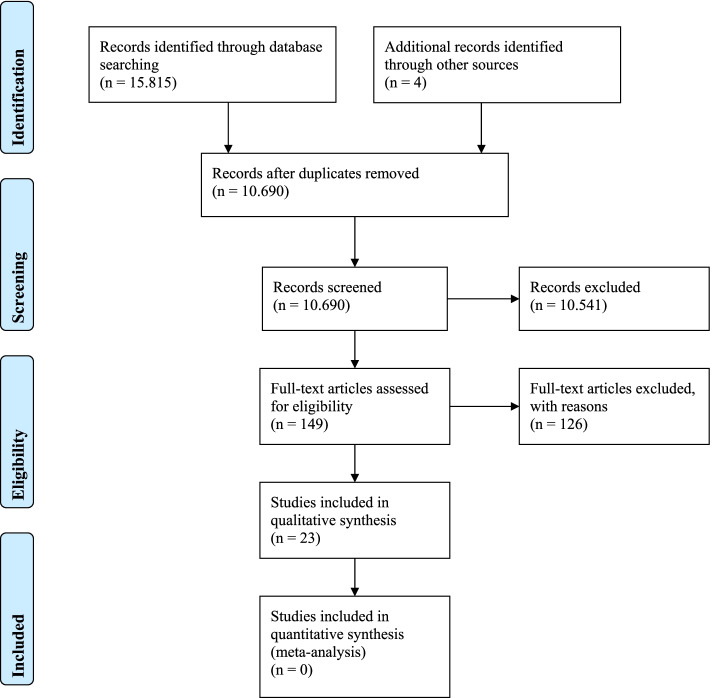
Prisma Flow Diagram (Moher, Liberati, Tetzlaff, Altman, & Prisma Group, 2009) [18]

The databases search resulted in 15,815 records, four from chain searching. After duplicates were removed, the total was 10,690 unique records. 10,541 of these were excluded in title and abstract screening, leaving 149 for full-text screening. Of those, 126 were excluded, primarily for design and population reasons, and 23 studies were qualitatively synthesised. Even through the inclusion criteria permit studies from 1996, the included studies date from 2001 to 2019 (Fig. 1).

### Characteristics of sources of evidence

Table 1 shows study characteristics.

**Table 1:** Study characteristics

Author and year	Country and setting	Population	Sample size (***n***)	Mean age and sex	Treatment period	Design	Cannabis delivery	Cannabis content	Tests	Outcome	Findings	Possible affected cognitive functions	EPHPP
**Sativex spray**^**a**^													
Alessandria, 2020 [9]	Italy. University of Genoa	MS	20	Age: 50.2 ± 11.4 Female/male: 11/9	12 months (including titration)	Pre-post study	Sativex spray	THC, 27 mg/mL CBD, 25 mg/mL Self-titration over 2 weeks. Average = 5/day range 2–9 Max dose 12 sprays/day.	The Italian version of the International Cognitive Assessment for Multiple Sclerosis (BICAMS): Symbol Digit Modalities Test (SDMT) California Verbal Learning Test (2. Version) (CVLT) The Brief Visuospatial Memory Test (BVMT-R) PASAT The Free and Cued Selective Remind Test (FCSRT)	Cognition	**Improvement** Improvement 6-MO: SDMT: 2.5, *p* < .001. CVLT: 5.7, *p* < .0001 Improvement 12-MO: SDMT: 2.0, *p* = .020. CVLT: 7.0, *p* < .0001 Corrected for multiple testing	Visual scanning, mental flexibility, sustained attention, psychomotor speed, and speed of information processing Learning and memory, semantic clustering, intrusions, interference, and recognition	Weak
Aragona, 2009 [22]	Italy. MS Outpatient Clinic	MS	17	Age: 49.8 ± 6.64 Female/male: 11/6	3 weeks (including titration) with 2 weeks washout between active periods	Randomised, double-blind, placebo-controlled, crossover	Sativex spray	Mean puffs/day: Active group: 8.20, SD = 3.15 Placebo group: 15.16, SD = 4.51	Paced Auditory Serial Additional Test (PASAT)	Fatigue, disability, cognitive functioning and quality of life	**N.S.**		Strong
Cooper, 2017 [23]	UK. King's College London	ADHD	30	Active group (*n* = 15): Age: 36.91 ± 11.70. Female/males: 6/9 Placebo group (n=15): Age: 38.90 ± 11.54 Females/males: 5/10	42 days (excluding 2 weeks titration)	Two-group, randomised, double-blind, placebo-controlled	Sativex spray	Maximum 14 sprays/day. Mean number of active sprays/day = 4.7, SD = 3.3	Quantitative Behavioural Test (QbTest) (Sustained attention and response inhibition, as well as motor restlessness) The Sustained Attention to Response Task (SART)	Cognitive performance and activity level (head movements) measured using the QbTest	**N.S.**		Moderate
Rog, 2005 [14]	UK. Walton Centre Clinical Trials Unit	MS + neuropatic pain	66 34 active group	Active group (*n* = 34): Age: 50.3 ± 6.7 Female/male: 28/6 Placebo group (*n* = 32): Age: 48.1 ± 9.7 Female/male: 24/8	5 weeks, 4 visits (including titration)	Two-group randomised, double-blind, placebo-controlled	Sativex spray	Maximum 48 sprays/day. Mean number of sprays/day: Active group = 9.6. SD = 6.0 Placebo group = 19.1 SD = 12.9	Spatial Recall Test Symbol Digit Modalities Test Paced Auditory Serial Addition Test Word Generation List Selective Reminding Test	Primary: pain Secondary: sleep disturbance, neuropsychological outcomes, anxiety, depression and disability.	**N.S.**		Strong
Vachová, 2014 [15]	Czech Republic. Six centres	MS + spasticity	121 61 active group	Age: 48.6 ± 9.64 Female/male = 76/45	48 weeks (excluding 2 weeks titration)	Two-group randomised, double-blind, placebo-controlled	Sativex spray	Maximum 12 sprays/day. Mean number of sprays 6–8 in active group	PASAT	Primary: Cognition Secondary: subject-, physician- and caregiver global impression of change	**N.S.**		Strong
Wade, 2004 [24]	UK. Three clinical centres	MS	160	Active group (*n* = 80) Age: 51.0 ± 9.4 Female/male: 47/33 Placebo group: (*n* = 80) Age: 50.4 ± 9.3. Female/male: 52/28	6 weeks	Two-group randomised, double-blind, placebo-controlled	Sativex spray	Maximum of 120 mg THC and 120 mg CBD per day and < 20 mg of each in any 3-h period.	Short Orientation- Memory-Concentration Test Adapted Adult Memory and Information Processing Battery Test of Attention.	Primary: symptoms in multiple sclerosis Secondary: Other symptoms, disability, cognition, mood, sleep and fatigue	**N.S.**		Strong
Russo, 2016 [25]	Italy. IRCCS Centro Neurolesi “Bonino-Pulejo”, Messina	MS + spasticity	61	Age: 43 ± 9 Female/male: not reported	6 months	Pre-post	Sativex spray	The number of sprays was gradually augmented within 10 days up to 8–9 puffs	Montreal Cognitive Assessment (MoCA), The Attentive Matrices (AM), The Trail Making Test (TMT-A, B, BA), The Babcock Story Recall Test (BSRT), RT Hand Motor Task	Spasticity and cognition	**N.S.**		Moderate
Castelli, 2018 [26]	Italy. Setting not reported	MS	22 Continuers = 11 Quitters = 11	Age: 49.7 ± 8.3 Female/male: 13/9	1 year	Case-control, groups are studied retrospective, continuers vs. quitters	Sativex spray	Median sprays/day in both groups = 6	The Stroop Color-Word Test	Primary: postural sway with and without dual task (Stroop)	**Impairment** Dual task condition *F*[1.9, 37.4] = 1.19, *p* = 0.312, η2 = 0.06	Ability to inhibit cognitive interference, attention, processing speed, cognitive flexibility, and executive function	Weak
Epidiolex^b^													
Martin, 2019 [17]	USA. University of Alabama	Treatment resistant epilepsy.	27	Age: 34 ± 14 Female/male: 14/13	1 year (including titration)	Pre-post	Epidiolex (highly purified CBD) Oral solution 100 mg/ml	Start dose: 5 mg/kg/day (divided between morning and evening). Maximum dose: 50 mg/kg/day. Mean dose: 36.5 mg/kg/day	NIH Toolbox: Dimensional Change Card Sort (DCCS), Flanker Inhibitory Control and Attention Test, Picture Sequence Memory Test, Pattern Comparison Processing Speed Test, List Sorting Working Memory Test, Oral Reading Recognition Test, The Picture Vocabulary Test	Primary: Cognitive functioning	**N.S.** Bonferroni’s corrected for multiple testing		Weak
Metternich, 2020 [27]	Germany. University of Freiburg	Pharma-coresistant epilepsy	13 (Adults completers)	Age: range 18–59 years Female/male: not reported	Three months (including titration)	Pre-post	Epidyolex/Epidiolex (*N* = 10) or Pharmaceutical formulation of synthetic CBD in a 100 mg per ml MCT-oil-based oral solution (*N* = 38)	Epidyolex: 100 mg CBD per 1 ml 2–5 mg/kg/day divided into two daily doses and triated up to 18–20 mg/kg/day over 14–21 days	Verbal Learning and Memory Test (VLMT) Digit Span Semantic verbal fluency Five point test Trail Making Test (TMT A & B) D2 The Epitrack ®: TMT-A, TMT-B, a maze task, a phonemic verbal fluency task and a measure of digit span backwards (Incl. parallel version) semantic verbal fluency (animals or food) Design fluency (the five-point-test) Auditory Verbal Learning Test	Primary: Cognitive and behavioral	**N. S.**		Moderate
Other spray													
Almog, 2020 [28]	Israel. Pain Research Unit of Rambam Health Care Campus, Haifa	Chronic pain	27 (ITT = 25)	Age: 48.3 ± 11.9 Female/male: 8/19	3 doses on 3 separate days (3×3)	Three-group randomised, double-blind, placebo-controlled, crossover	Aerosolize doses Syqe Inhaler, software controlled thermal selective-dose inhalation medical device.	Aerosolised doses of granulated raw plants 22% THC, < 0.1% cannabidiol (CBD), < 0.2% cannabinol (CBN) or a matched placebo 0.50 mg session: THC: 0.537 ± 0.052 mg 1.00 mg session: THC: 1.083 ± 0.076 mg	Cambridge Neuropsychological Test Automated Battery (CANTAB): Reaction Time Test, (RTI) Paired Associates Learning Task, (PAL) Spatial Working Memory Test, (SWM) Rapid Visual Information Processing Test, (RVP).	Primary: Analgesic efficacy Secondary: Safety and tolerability Cognitive	**N. S.**		Strong
Wade, 2003 [29]	UK. Outpatient clinics	MS (14) Spinal cord injury (4) Brachial plexus damage (1) Limb amputation due to neuro-fibromatosis (1)	20	Age: 48 Female/male: 10/10	2 weeks (including titration)	Randomised, double-blind, placebo-controlled, crossover	Spray (pump-action sublingual spray that delivered 2.5 mg THC and/or CBD at each actuation.)	Three types of concentrations: THC-rich CBD-rich 1/1 (THC/CBD) Max dose 120 mg/24 h	Short Orientation-Memory-Concentration Test	Intractable neurogenic symptoms, i.e. pain, spasticity.	**Impairment** THC-group: short orientation-memory-concentration test: 25.7, SD 3.4, *p* < 0.05.	Orientation, memory, and concentration	Moderate
Gelatin capsules													
Müller-Vahl, 2001 [30]	Germany. Outpatient clinic	Tourette syndrome patients	12	Age: 34 ± 13 Female/male: 1/11	Single dose Separated by 4 weeks washout period	Randomised, double-blind, placebo-controlled, crossover	Gelatin capsules 2.5 and 5.0 mg THC	Dose according to body weight, sex, and prior use of marijuana. A single dose of 5, 7, 5 and 10 mg.	Auditory Verbal Learning Test (VLMT), Digit Span, Multiple Choice Vocabulary test, Benton Visual Retention Test, Signal Detection, Vienna Reaction Time, Sustained Attention, Divided Attention.	Neuropsychological performance	**N.S.**		Moderate
Müller-Vahl, 2003 [31]	Germany. Outpatient clinic	Tourette syndrome patients	24 9 active group	Age: 33 ± 11 Female/male: 5/19	6 weeks (including titration)	Randomised, double-blind, placebo-controlled	Gelatin capsules 2.5 mg and 5.0 mg THC	*n* = 6, 10 mg *n* = 2, 7, 5 mg *n* = 1, 2, 5 mg	Auditory Verbal Learning Test Benton Visual Retention-Test Divided Attention (TAP) Multiple Choice Vocabulary Test	Neuropsychological performance	**N.S.**		Moderate
Vaney, 2004 [16]	Switzerland. In-patient rehabilitation centre	MS + spasticity	57 50 Intention-to-treat analysis	Age: 54.9 ± 10 Female/male: 29/28	14 days (excluding titration)	Randomised, double-blind, placebo-controlled, crossover	Gelatin capsule whole plant extract	Capsule: THC: 2.5 mg, CBD: 0.9 mg Maximum of 30 mg THC/day	Paced Auditory Serial Addition Test (PASAT) Digit span (WAIS R)	Primary: efficacy, safety and tolerability in the treatment of spasticity Secondary: daily activities cognitive functions	**N.S.**		Strong
Smocked or vaped													
Corey-Bloom, 2012 [11]	USA. MS clinic	MS + spasticity	30	Age: 51 ± 8 Female/male: 19/11	3 days	Randomised, double-blind, placebo-controlled, crossover	Smoked	4 puffs of pre-rolled cannabis cigarettes contained about 4% THC by weight	Paced Auditory Serial Addition Test (PASAT)	Primary: change in spasticity Secondary: pain, physical performance, cognitive function	**Impairment** PASAT: Reduction of 8.67 points (95% bootstrap CI 4.10 to 14.31) more than placebo (*p* = 0.003)	Attention, sustained attention, working memory, and processing speed	Moderate
Wallace, 2015 [12]	USA California Outpatient clinic	Painful diabetic peripheral neuropathy	16	Age 56.9 ± 8.2 Female/male: 7/9	3 h Separated by 2 weeks washout periods	Randomised, double-blind, placebo-controlled, crossover	Cannabis cigarette Aerosolized Volcano system vaporizer	Three types of concentrations: 1% THC 4% THC 7% THC CBD concentration was < 1%. 0, 4, 16, or 28 mg THC per dosing session.	Trail Making Test A and B, Paced Auditory Serial Attention Test (PASAT).	Pain, evoked pain, and cognitive	**Impairment** PASAT: medium dose at 15 min: *d* = 1.03, *p* = .024) high dose at 15 min: *d* = − 1.14, *p* = .008 Trail making part B: high dose at 120 min: *d* = − 1.15, *p* = .009 Adjusted for multiple comparisons using the Dunnett method	Attention, sustained attention, working Memory, and processing speed	Strong
Wilsey, 2008 [13]	USA California Davus/Sacramento VA medical Center	Neuropathic Pain patients	38	Median age: 46 Range 21–71 Female/male: 18/20	Three 6-h sessions Separated by 3 days washout periods	Randomised, double-blinded, placebo controlled, crossover	Smoked cannabis cigarettes	Cannabis ranging in strength from 3 to 7% THC THC 19 mg low dose THC 34 mg high dose	Wechsler Adult Intelligence Scale (WAIS-III), Digit Symbol Test, The Hopkins Verbal Learning Test Revised (HVLT), The Grooved Pegboard Test	Primary: pain intensity Secondary: neuropsychological performance	**Impairment** Mean Difference: pegboard dominant hand: 7% vs placebo: 1.14, *p* = .007 pegboard non-dominant hand: 7% vs placebo: 1.34, *p* < .001 3.5% vs placebo: 1.01, *p* < .01 Last point vs linear trend: 3.19, *p* < .01 Digit symbol test: last point vs linear trend: 1.30, *p* = .001 HVLT–learning: 7% vs placebo: 1.31, *p* = .02 Last point vs linear trend: 6.19, *p* < .001 HVLT–recall: 7% vs placebo: 1.30, *p* = .03 Last point vs linear trend: 6.16, *p* < .000 Not corrected for multiple testing	Visual scanning, mental flexibility, sustained attention, psychomotor speed, and speed of information processing, verbal learning and memory, psychomotor speed, fine motor control, and rapid Visual-motor coordination	Strong
Wilsey, 2013 [32]	USA California Pain clinics	Neuropathic pain patients	39	Age: 50 ± 11 Female/male: not reported	Three 6-h sessions, separated by 3 days washout periods.	Randomised, double-blind, placebo-controlled, crossover	Vaporised Volcano vaporiser	Cannabis ranging in strength from 1.29 to 7% THC Medium-dose (3.53% THC), 19 mg Low-dose (1.29% THC), ca. 9.5 mg	Wechsler Adult Intelligence Scale (WAIS-III), Digit Symbol Test, The Hopkins Verbal Learning Test Revised (HVLT), The Grooved Pegboard Test	Primary: spontaneous pain relief Secondary: neurocognitive	**Impairment** Significance levels: pegboard dominant hand: 60 min: *p* = .0007, 240 min: *p* = .0023 Pegboard non-dominant hand: 120 min: *p* = .0035, 180 min: *p* = .0325 WAIS III digit symbol: 60 min: *p* = .0415, 180 min: *p* = .0006 HVLT sum of all trials: 60 min: *p* = .0256, 180 min: *p* < .0001, 240 min: *p* = .0002 HVLT delay: 120 min: *p* = .0273, 180 min: *p* = .0013, 240 min: *p* = .0060 Not corrected for multiple testing (Only significance levels are reported)	Visual scanning, mental flexibility, sustained attention, psychomotor speed, and speed of information processing, verbal learning and memory, psychomotor speed, fine Motor control, and rapid visual-motor coordination	Strong
Abdallah, 2018 [33]	Canada. McGill University	Global Initiative for Obstructive Lung disease	16	Age: 65.4 ± 7.7 Female/male: 6/10	Single dose	Randomised, double-blind, placebo-controlled, crossover	Vaped	THC 35 mg, 18.2% CBD < 0.1%	Mini-Mental Status Examination (MMSE)	Primary: breathlessness intensity ratings during exercise exercise endurance time	**N.S.**		Moderate
Other													
Bar-Sela, 2019 [34]	Israel. Day Care Clinic	Advanced cancer patients	34	Active group (*n* = 17): Age: 63, range 35–85 Female/male: 10/7 Control group (*n* = 17): Age: 63, range 40–85 Female/male: 7/10	3 months	Case-control	Own choice: smoking, inhalation or oil.	Three types of concentrations (THC/CBD): 1/1, *n* = 1 2/1, *n* = 4 3/1, *n* = 12	The Montreal Cognitive Assessment (MoCA), Digit Symbol Substitution Test (DSST), Digit-Finger Tapping Test	Primary: cognitive function secondary: symptom relief and QOL	**N.S.**		Weak
Gustavsen, 2021 [35]	Denmark. University Hospital, Copenhagen	MS	28	Age: 50 (range 27–74) Female/male 21/7	Four weeks (including titration)	Pre-post	Three full spectrum oils from the company STENOCARE, Denmark	Full-spectrum cannabis extracts: THC DROPS (25 mg THC, < 2 mg CBD/mL) CBD DROPS (25 mg CBD, 2 mg THC/mL) 1:1 DROPS (12.5 mg THC and CBD/mL) + due to production stop Broad spectrum cannabis product: THC/CBD 1:2.5.^c^	9-Hole Peg Test (9-HPT) Symbol Digit Modalities Test (SDMT)	Primary: adverse events changes in neurological examination Secondary: treatment effectiveness	**N.S**.		Moderate
Withdrawal													
Feinstein, 2019 [10]	Canada Sclerosis clinic	MS	39 Cannabis continuation (CC) = 20 Cannabis withdrawal (CW) = 19	CC group: Age: 39.3 ± 8.47 Female/male: 9/11 CW group: Age: 36.26 ± 11.69 Female/male: 11/8	28 days withdrawal CW mean = 5.62 years (SD = 5.10) versus CC mean = 9.61 years (SD = 5.67), *t* = 2.31; *P* = 0.03].	Randomized, controlled MS patients and cannabis users were divided by odd-even number selection into two groups: cannabis continuation and cannabis withdrawal. Controlled by urine test.	–	From urine tests: THC/creatinine ratio CC = 165.65 day 28.	Brief Repeatable Neuropsychological Battery (BRNB) for Multiple Sclerosis Functional: The Selective Reminding Test, the 10/36 Test, Paced Auditory Serial Addition Test (PASAT), Symbol Digit, Modalities Test (SDMT), Controlled Oral Word Association Test (COWAT)	Primary: cognition	**Improvement with withdrawal** PASAT 3”: *t* = − 7.85, *p* < .000 PASAT 2”: *t* =− 7.48, *p* < .000 SDMT: *t* = − 5.10, *p* < .000 COWAT: *t* = − 4.22, *p* < .000 Controlled for multiple comparisons	Attention, sustained attention, working memory, processing speed, visual scanning, mental flexibility, psychomotor Speed, speed of information processing, and verbal fluency	Weak

*Abbreviations: MS* multiple sclerose, *ADHD* attention deficit and hyperactivity disorders, *ITT* intention to treat

^a^Sativex spray: Each actuation delivers 100 μL of spray, containing THC 2.7 mg and CBD 2.5 mg

^b^Epidiolex: Oral solution containing 100 mg cannabidiol

^c^Unfortunately, 6 months after study initiation the supplier of the three full-spectrum oils, CannTrust, Canada, was involved in a case with the Canadian authorities for using unapproved cultivating grow rooms. As a result, STENOCARE immediately stopped the import and distribution of their products. Subsequently, one pharma-grade broad-spectrum cannabis product (THC/CBD, 1:2.5) was available, which contained active pharmaceutical Ingredients (API) of both THC and CBD. The API of THC and other organic compounds were extracted from dried cannabis flowers, Bedrocan (high THC, low CBD) by solvent extraction

Twenty-three studies examined the effect of CBMs on cognitive functioning within the inclusion criteria. The studies comprised a total of *N* = 917. The mean age ranged from 33 to 65 years for 448 females and 356 males (three studies did not report sex). The sample size ranged from 11 to 160. The study populations were as follows: multiple sclerosis (MS) (*n* = 11), neuropathic pain (*n* = 2), Tourette’s syndrome (*n* = 2), treatment-resistant epilepsy/pharmaco-resistant epilepsy (*n* = 2), obstructive lung disease (*n* = 1), advanced cancer (*n* = 1), attention deficit hyperactivity disorder (ADHD) (*n* = 1), painful diabetic peripheral neuropathy (*n* = 1), chronic pain (*n* = 1) and a mix of pain patients (MS, spinal cord injury, brachial plexus damage and limb amputation due to neurofibromatosis) (*n* = 1).

Fifteen studies reported non-significant findings, six reported impairment of the cognitive functioning, one study found improvement and a single study found improvement with withdrawal. In total, fifteen studies were randomised, double-blind, placebo-controlled; one randomized controlled, one case-controlled and six pre-post studies. Four studies had a treatment period of a single day, one 3 days and the remaining studies between 2 weeks and 12 months.

In total, eight studies used Sativex in their treatments. Two studies used Epidiolex, and one study used a spray (SyqeAir Inhaler). Three studies used gelatine capsules and three studies used smoking/vaping for cannabis delivery.

Fourteen studies had cognitive/neuropsychological functioning as their primary outcome. The most common tests used were the Paced Auditory Serial Addition Test (PASAT), Digit Symbol Substitution Test (DSST), Trail Making Test (TMT) and Verbal Learning Test (VLT).

Fourteen studies were from Europe, seven from North America and two from Israel.

Nine studies received a strong rating on the modified EPHPP, nine a medium rating and five a weak rating. A summary of global and component ratings of each study is provided in Table 2.

**Table 2:** Summary of global and component ratings of each study

	Selection bias	Study design	Blinding	Data collection method	Withdrawals and dropouts	Global rating
**Sativex spray**						
Alessandria, 2020 [9]	Moderate	Moderate	Weak	Strong	Weak	Weak
Aragona, 2009 [22]	Moderate	Strong	Moderate	Strong	Strong	Strong
Cooper, 2017 [23]	Weak	Strong	Moderate	Strong	Strong	Moderate
Rog, 2005 [14]	Moderate	Strong	Moderate	Strong	Strong	Strong
Vachová, 2014 [15]	Moderate	Strong	Moderate	Strong	Strong	Strong
Wade, 2004 [24]	Moderate	Strong	Moderate	Moderate	Strong	Strong
Russo, 2016 [25]	Moderate	Moderate	Weak	Strong	Strong	Moderate
Castelli, 2018 [26]	Weak	Moderate	Weak	Strong	Weak	Weak
**Epidiolex**						
Martin, 2019 [17]	Moderate	Moderate	Weak	Strong	Weak	Weak
Metternich, 2020 [27]	Moderate	Moderate E	Weak	Strong	Strong	Moderate
**Other spray**						
Almog, 2020 [28]	Moderate	Strong	Moderate	Strong	Strong	Strong
Wade, 2003 [29]	Weak	Strong	Moderate	Moderate	Strong	Moderate
**Gelatin capsules**						
Müller-Vahl, 2001 [30]	Weak	Strong	Moderate	Strong	Strong	Moderate
Müller-Vahl, 2003 [31]	Weak	Moderate	Moderate	Strong	Strong	Moderate
Vaney, 2004 [16]	Moderate	Strong	Moderate	Strong	Moderate	Strong
**Smocked or vaped**						
Corey-Bloom, 2012 [11]	Weak	Strong	Moderate	Strong	Strong	Moderate
Wallace, 2015 [12]	Moderate	Strong	Moderate	Strong	Strong	Strong
Wilsey, 2008 [13]	Moderate	Strong	Moderate	Strong	Strong	Strong
Wilsey, 2013 [32]	Moderate	Strong	Moderate	Strong	Strong	Strong
Abdallah, 2018 [33]	Moderate	Strong	Moderate	Weak	Strong	Moderate
**Other**						
Bar-Sela, 2019 [34]	Weak	Moderate	Weak	Strong	Weak	Weak
Gustavsen, 2021 [35]	Moderate	Moderate	Weak	Strong	Strong	Moderate
**Withdrawal**						
Feinstein, 2019 [10]	Weak	Strong	Weak	Strong	Strong	Weak

### Synthesis of results

The included studies turned out to be extremely heterogeneous in design, populations and treatment period. To provide a better overview, the studies were divided into seven groups according to how the cannabis treatment was delivered: Sativex spray, Epidiolex spray, other sprays, gelatine capsules, smoked or vaped, other delivery methods and withdrawal (see Table 1).

### Sativex

Sativex is a spray containing delta-9-tetrahydrocannabinol cannabidiol in a liquid carbon dioxide solvent. It contains 27 mg delta-9-tetrahydrocannabinol and 25 mg cannabidiol. Each single 100 μL spray contains 2.7 mg delta-9-tetrahydrocannabinol (THC) and 2.5 mg cannabidiol (CBD) from cannabis sativa L. Each spray also contains up to 40 mg ethanol [36].

Of the eight studies using Sativex, six of them showed non-significant results, one showed improvements and one showed impairment. Alessandria et al. [9] found stable improvements on the Symbol Digit Modalities Test (SDMT) and California Verbal Learning Test (2nd version) (CVLT). They treated 20 MS patients with Sativex (Nabiximols in the USA) to assess the long-term effects on cognition, mood and anxiety. The dosing was an average of 5 puffs per day, ranging from 2 to 9. Nine of the patients were defined as cognitively preserved and the 11 others as being impaired, defined by inadequate performances in at least two tests. Their results showed an improvement in one of three tests assigned to measure processing speed: the Symbol Digit Modalities Test (SDMT), after 6 months: visit 2 (V2)–visit 1 (V1) = 2.5; *p* < 0.001, min–max − 2.0–11.0 and after 12 months: V3–V1 = 2.0; *p* < 0.001, min–max − 2.0–20.0 and also improvement in a test assigned to measure auditory verbal memory (the California Verbal Learning Test) after 6 months: V2–V1 = 5.7; *p* = 0.0001, min–max − 7.0–20.0 and after 12 months: V3–V1=7.0; *p* = 0.020, min–max − 5.0–11.0. While both are statistically significant, there is no report as to whether they can be ascribed to a few major improvements as the range might imply.

Castelli et al. [26] tested the participants on a single task condition (the Stroop Color-Word Test) and a dual task condition (a postural task and the Stroop Color-Word Test simultaneously). They found impairment, but only in a dual task condition on a postural sway and the Stroop task. Their study was a small (*N* = 22) retrospective case-control study where quitters (discontinuation of CBMs) constituted the control group. The population consisted of MS patients treated with Sativex with a median daily dose of six puffs. Patients were tested at 1, 3 and 12 months on the Stroop Color-Word Test. There was only a significant decrease when the Stroop test was combined with a postural sway test at 12 months (*F*[2.6, 52.5] = 3.17, *p* = 0.038; η2 = 0.14) where scores decreased significantly (*p* = 0.025 by the post hoc Bonferroni test).

### Epidiolex (USA)/Epidyolex

Epidyolex is an oral solution containing 100 mg cannabidiol, 79 mg anhydrous ethanol, 736 mg refined sesame oil and 0.0003 mg benzyl alcohol per ml. The recommended starting dose of cannabidiol is 2.5 mg per kilogram body weight given twice a day (5 mg/kg/day) for 1 week. After 1 week, the dose should be increased to a maintenance dose of 5 mg/kg twice a day (10 mg/kg/day). Based on individual clinical response and tolerability, each dose can be further increased in weekly increments of 2.5 mg/kg administered twice a day (5 mg/kg/day), up to a maximum recommended dose of 10 mg/kg twice a day (20 mg/kg/day). It is dosed using syringes suppled in a carton [37].

Both studies using Epidyolex showed non-significant results. Both of them had cognition as the primary outcome and used a broad cognitive test battery.

### Other spray

Two studies used other sprays. Wade et al. [24] used a pump-action sublingual spray delivering 2.5 mg THC and/or CBD at each actuation. They used three combinations of THC and CDB ratios: THC-rich, CBD-rich and one-to-one. The study assessed 20 participants with a consecutive series of double-blind, randomised, placebo-controlled cross-over trials, each with a 2-week treatment period. The study used The Short Orientation-Memory-Concentration Test of Cognitive Impairment as the cognitive test. The test is a six-item test with questions such as ‘what year is it’ and ‘say the months in reverse order’. The THC group had a significant drop in score from baseline. The baseline was 27.1 (1.9) and the THC group was 25.7 (3.4). The maximum score is 28, and the high baseline score indicates a risk of sealing effects, which makes the test less sensitive to detecting cognitive impairment. It is unclear when the dose was administered and hence whether the patients were intoxicated by THC while being tested.

Almog et al. [28] used the Syqe Inhaler to test the pharmacokinetics, analgesic effect, cognitive performance and safety effects in patients with chronic pain. The Syqe Inhaler is a software-controlled, thermal selective-dose inhalation medical device. In this study, it delivered aerosolised doses of granulated raw plants 22% THC, < 0.1% cannabidiol (CBD), < 0.2% cannabinol (CBN) or a matched placebo. During three sessions, 25 patients received a single inhalation of THC: 0.5 mg, 1 mg, or a placebo. The patients were tested on selected tests from the Cambridge Neuropsychological Test Automated Battery (CANTAB). Following the Reaction Time Test (RTI) and Rapid Visual Information Processing Test (RVP), improvement was seen after 15 min at 0.5 mg. The Spatial Working Memory Test (SWM) showed an impairment after 15 min with 1.0 mg. None of these indications were supported by neither significant time-by-dose interactions or an effect of dose in the analysis of variance, nor correlation between plasma concentration and cognitive performance, and all three results turn non-significant after Bonferroni correction for multiple testing.

### Gelatine capsules

In two studies by Muller-Vahl et al. [30] and Müller-Vahl et al. [31], patients with Tourette’s syndrome were treated with gelatine capsules with single doses of 5, 7.5 and 10 mg THC in randomised, double-blind, placebo-controlled studies and tested on a broad cognitive test battery with non-significant results. Likewise, Vaney et al. [16] tested MS patients with gelatine capsules containing whole plant extracts and THC 2.5 mg; CBD 0.9 mg—with a maximum of 30 mg THC/day on three cognitive test and found no significant results.

### Smoked or vaped

All four studies that delivered cannabis by smoking or vaping showed significant impairments. They had also all relatively high doses and relatively short treatment periods.

Corey-Bloom et al. [11] tested 30 MS patients on the PASAT about 45 minutes after smoking four puffs from an 800 mg cannabis cigarette. This gave a reduction in performance compared to the baseline by 8.67 points more than placebo on the PASAT (95% bootstrap CI 4.10 to 14.31, *p* = 0.003). However, the reduced scores were still ‘within normal ranges for their ages and levels of education’. Also, Wallace et al. [12] found impaired performance shortly after 16 patients with painful diabetic peripheral neuropathy had inhaled vaporised cannabis—however, only with the medium and high doses of 16 mg and 28 mg THC, respectively, pr. session. The participants was tested using the PASAT and Trail Making A and B tests. The patients showed significantly decreased performance from PASAT (16 mg (*d* = − 1.03, *P* = .024) and 28 mg (*d* = − 1.14, *P* = .008) at 15 min) and Trail Making B (28 mg, only at 120 min (*d* = − 1.15, *P* = .009))—however, not enough to enter the impairment range and only between 15 and 120 min post-cannabis exposure. Note: A secondary analysis [38] showed a significant linear effect of THC on PASAT, suggesting that higher THC levels were associated with decreased cognitive performance on this test, *P* = .002, and this effect surpassed a stringent Bonferroni adjustment.

Similarly, Wilsey et al. [13] found that a high dose of 34 mg of THC significantly impaired cognitive performance within 6 h of exposure. Thirty-eight patients with central and peripheral neuropathic pain smoked cannabis equal to a low dose of 19 mg of THC or a high dose of 34 mg of THC. The high dose gave evidence of cognitive impairment in attention, learning and memory, and psychomotor speed, whereas the low dose resulted in a decline in learning and memory only.

Wilsey et al. [32] found comparable results since their medium dose of 19 mg (same dose as ‘low’ in Wilsey et al. [13] gave significant and low-to-medium effects on learning and memory within 2 h post-cannabis exposure, but none after 3 h. They found some inconsistent effects on psychomotor function. Only the low dose (9.5 mg) gave a significant effect on the dominant hand condition after one (*p* = .0007) and 4 (*p* = .0023) hours, but no significant effect after 2 and 3 h. The non-dominant hand condition was affected after 2 (*p* = .0035) and 3 (*p* = .0325) hours, but not at 1 and 4 h. The significant (but low) effects on verbal learning manifested themselves with both low and medium doses, but were inconsistent over time.

Abdallah et al. [33], used a randomised, double-blind, placebo-controlled crossover design to test patients with Obstructive Lung Disease. The primary outcome was difference in breathlessness intensity ratings during exercise. The single dose of 35 mg vaporised cannabis to 16 participants did not result in a significant difference on cognition measured using the Mini Mental State Examination (MMSE), a screening test for dementia with a high risk of sealing effects on this population (scores above 29 out of 30).

### Other

Two studies were included which used other ways of delivering cannabis.

Bar-Sela et al. [34] tested 34 patients with advanced cancer during chemotherapy treatment over a period of 3 months. The participants received CBM with ratios of 1:1, 1:2 or 1:3 CDB:THC by their preferred way—vaped, smoked or oil. The primary outcome was cognitive functioning assessed with the MoCA, Digit Symbol Substitution Test (DSST) and Digit-Finger Tapping Test at 3 months. On the DSST, improvements in learning effects were seen in both groups, and there were no significant differences between the groups.

Gustavsen et al. [35] did find improvement in MS patients with full-spectrum cannabis oils in the 9-HPT test (dominant hand), median diff − 1.7 s, *p* < .01. However, because it was a test of dexterity and the median differences were minor, the improvement was not considered clinically significant.

### Withdrawal

The potential impact of CBMs on cognitive functioning can also be studied by assessing the potential positive impact in cognitive performance after withdrawal from cannabis treatment. In a sample of 39 MS patients with long-term use of CBMs (average years 5.62, SD = 5.10), Feinstein et al. [10] showed significant improvements in memory, processing speed and executive function after 28 days of drug abstinence compared to matched controls with continued use, measured by PASAT 3”, *t* = − 7.85, *p* < .000 and PASAT 2”, *t* = − 7.48, *p* < .000, Symbol Digit Modalities Test (SDMT) *t* = − 5.10, *p* < .000, and the Controlled Oral Word Association Test (COWAT) *t* = − 4.22, *p* < .000. No significant change was found in the control group over time, apart from deteriorating visual memory. However, the studies should be interpreted with caution due to the selection bias from choosing long-time users and the lack of proper blinding that comes from participants knowing they do not take the drug anymore.

### Cognitive tests

At least 43 different tests were used in the included studies. Only five tests (the Symbol Digit Modalities Test, the Digit Symbol Test, PASAT, the Trial Making Test (TMT-A, B) and Digit Span) were used by more than one author group. All these tests measure some aspects of working memory or attention. The otherwise very diverse selection of tests makes it exceedingly difficult to compare studies and effects of cannabis on other cognitive domains. However, to ease the interpretation an overview of the tests with significant findings and the potential cognitive functions measured is presented below (see also Table 1).

### Tests and cognitive functions

Few studies have explicitly stated hypotheses about cognitive functions to be affected by CBMs, hence one should be cautious in the interpretation of the findings.

The California Verbal Learning Test measures verbal learning and memory and in addition attempts to measure a broad range of cognitive functions such as free and cued recall, serial position effects (including primacy and recency), semantic clustering, intrusions, interference and recognition [39].

The Controlled Oral Word Association Test is a verbal fluency test that measures spontaneous production of words belonging to the same category or beginning with some designated letter [40].

The Grooved Pegboard Test measures psychomotor speed, fine motor control and rapid visual-motor coordination [41].

The Hopkins Verbal Learning Test Revised is a brief verbal learning and memory test [42].

The Paced Auditory Serial Addition Test (PASAT) is often used to assess attention and concentration [41]. Also, it has shown clinical utility in detecting impairments in cognitive processing in a wide variety of neuropsychological syndromes [41]. It is recognised as a measure of multiple functional domains, primarily those related to attention, but also sustained attention, working memory and processing speed [43].

The Short Orientation-Memory-Concentration Test is a 6-item mental status questionnaire/test that have shown to be useful as a screening [44].

The Stroop Color and Word Test is often used for assessing the ability to inhibit cognitive interference but also other cognitive functions such as attention [43], processing speed, cognitive flexibility [45] and Executive function [46].

Substitution tests (Digit Symbol Substitution Test & Symbol Digit Modality Test) are, like the Trail Making Test sensitivity to the presence of cognitive impairment and often used as a component of screening batteries sensitive to brain dysfunction [47] since they draw on many different processes, including visual scanning, mental flexibility, sustained attention, psychomotor speed and speed of information processing [48].

The Trail Making Test is popular due to its high sensitivity to the presence of cognitive impairment often used as a component of screening batteries [41]. A comprehensive review of the literature by Sánchez-Cubillo et al. [49] showed Trail Making tests are used for assessing working memory, inhibition/interference control, task-switching ability and visuomotor speed and that Part-B reflects primarily working memory and secondarily task-switching ability, while B-A minimises visuoperceptual and working memory demands, providing a relatively pure indicator of executive control abilities.

### Risk of bias

The most common ground for a weak retention was ‘selection bias’ and ‘blinding’, with eight studies each. Four studies were rated as *weak* in withdrawals and dropouts’ and the same applied to one study in data collection method’.

## Discussion

The aim of this systematic review was to uncover the field for the potential impact of CBMs on cognitive functioning when used in a controlled setting as part of the treatment for chronic pain and other medical conditions. In total, 23 studies were included. The studies were vastly different in both design and populations included. Fifteen of the twenty-three studies found no significant impact of CBMs on cognitive functioning, but six studies did find that CBMs had an adverse impact on cognition. Although the impaired cognitive functioning was found to be significant, all the test scores were either within the normal range or below what would be characterised as a neuropsychologically cognitive impairment. Moreover, impairment was only seen in relatively short periods after high doses of THC when patients presumably were intoxicated during testing. Furthermore, most of the studies that found impaired cognitive functioning were characterised by being short single-session treatments with cannabis delivery and cognitive testing-retesting within hours. For these reasons, the results cannot be generalised on patients undergoing long-term stable treatment with CBMs. It is important to differentiate between intoxication or withdrawal from prolonged abuse and controlled medical treatment without or with limited psychoactive effects. The former has been extensively studied and is known to affect cognitive functions, as also mentioned in the recent review by Landrigan et al. [7] whereas the latter has not been studied as extensively and thus is the subject of this review. Basically, we want to know if we can treat patients with CBM without negatively affecting, or affecting to a minimal degree, their cognitive functions and hence their daily activities. This includes examining potential effects after cessation, as in the study by Feinstein et al. [10], which found improved processing speed, memory and executive functioning after withdrawal from long-term use of CBMs, indicating that stable long-term use may adversely impact cognitive functioning. The results should be interpreted with caution, since no blinding was applied, and the sample size was small, reflected in a weak rating on the EPHPP. The included studies are very diverse in factors such as dose, duration, type of cannabis, route of administration, prior history and other drug(s) used. All of these are factors that presumably play a key role in examining potential adverse effects on cognition. The diversity makes any possible comparisons across studies very limited and hence hinder our understanding. There seems to be a trend towards using Sativex in more controlled settings and uniform patient groups. This will greatly enhance the evidence. Still, thought should be given as to how studies can incorporate prior use and other drug use in the design, as this reflects the real-life situation of many patients.

In agreement with the systematic review by Landrigan et al. [7], our results indicate that the impact of CBMs on cognitive functioning is minimal as long as the doses of THC are low to moderate. Unfortunately, the studies are too divergent to specify the maximum dose of THC tolerated before cognition is negatively affected. However, among patients with neuropathic pain, the two studies by Wilsey et al. [13, 32] indicate that treatment with a THC dose below 19 mg did not affect cognition significantly differently from the placebo group.

The results of the present review do not reflect the high number of adverse events (OR 5.67) in relation to ‘cognition and attention disturbances’ reported in the meta-analysis by Stockings et al. [6]. This may be due to the fact that reporting of adverse events in Stockings et al. [6] was based on self-reported symptoms, which is vastly different from objective test results on recognised neuropsychological tests administered in a controlled setting. This is an important finding, since impaired cognitive functioning associated with CBMs may result in patients rejecting the treatment on false premises due to fear of reduced cognitive functioning. But again, the heterogeneity of cognitive tests used across studies without population norms, combined with the vastly different study designs, patient populations and type of CBMs used, make it impossible to draw definite conclusions about the impact of CBMs on cognitive functioning.

## Methodological limitations of the included studies

Overall, a number of methodological limitations of the included studies need to be taken into account when interpreting the present findings. As already mentioned, the large heterogeneity in study design, patient populations, dose and type of CBMs used, as well as the cognitive tests applied, constitute a major limitation. Measurement of CBD/THC levels across studies is complicated by different delivery methods. For instance, four puffs of a cigarette with a given THC percentage tells us little about the THC levels at the time of cognitive testing, when time from intake to assessment is not reported. Without precise information on (at the very least) doses and time since intake, we are left without a proper way of assessing levels of intoxication and whether intoxication has affected the results on the cognitive tests. Milliseconds of either improvement or worsening on two different cognitive tests of attention is not comparable without population norms. Also, there is no consensus on a gold standard for cognitive tests for the different cognitive domains, and ceiling effects are achieved on the cognitive tests in several of the included studies. Another major limitation is test-retest effects. While some tests are more sensitive to this, the time span between testing is also an important factor to include. In particular, studies applying the same tests multiple times within a few minutes are prone to test-retest effects. These limitations are also partly reflected, although not completely captured, in the different ROB results across the included studies.

## Limitations of this review

Our review was, by design, broad in scope on both cognitive functions and CBMs. The purpose was to uncover the field, even at the risk of including studies that are difficult or impossible to compare, and this has indeed turned out to be the case. Another limitation were the choices made in order to handle the very broad scope in search strings. It was necessary to exclude terms, e.g. ‘concentration’ from the more medicine-based databases to filter out results concerning chemistry, at the risk of missing results concerning focused attention.

We have chosen to discriminate between non-cancer and cancer pain and only include studies of the former. Although this discrimination is debated, most of the research literature still uses this distinction why we also chose to do so in order to facilitate the interpretation of already complex results. That being said, the distinction may be somewhat unscientific since the two groups share pain generation physiologies. However, health behaviours, psychological comorbidities and use of opioids may differ due to the malign nature of cancer-related pain—and all of these are factors that further complicate interpretation of the potential impact of cannabis-based medicines on cognition.

A more relevant distinction in future studies could be according to the new ICD-11 diagnosis of primary and secondary pain. However, since this is a new distinction, it was difficult to apply in the current study.

## Conclusion

Due to large heterogeneity and methodological limitations across studies, it is not possible to make any definite conclusion about the impact of CBMs on cognitive functioning. However, the majority of high-quality evidence suggests that the negative impact of CBMs on cognitive functioning is small, as long as the doses of THC are low to moderate. On the other hand, long-term use of CBMs may still negatively influence cognitive functioning. The cognitive domains mostly found to be negatively affected by CBMs are attention/concentration and memory. No evidence of this review indicates that CBD severely influences cognitive functioning, at least not when taking the doses applied in the included studies. The potential positive effect of CBMs on cognitive functioning may be due to practice effects or mediated by alleviation of other medical symptoms, such as pain, depression or sleep problems. More high-quality longitudinal placebo-controlled studies assessing the potential long-term impact of CBMs on cognitive functioning are needed. Especially fundamental is the focus on CBMs for specific medical conditions with control for dose and type of CBMs, as well as the use of validated cognitive tests.

## Data Availability

All analysed data are included in this manuscript.

## Abbreviations

ADHD: Attention deficit hyperactivity disorder
AM: Attentive matrices
BICAMS: International Cognitive Assessment for Multiple Sclerosis
BRNB: Brief Repeatable Neuropsychological Battery
BSRT: Babcock Story Recall Test
BVMT-R: Brief Visuospatial Memory Test
CANTAB: Cambridge Neuropsychological Test Automated Battery
CBD: Cannabidiol
CBMs: Cannabis-based medicines
CBN: Cannabinol
COWAT: Controlled Oral Word Association Test
CVLT: California Verbal Learning Test
DCCS: Dimensional Change Card Sort
DSST: Digit Symbol Substitution Test
EPHPP: Effective Public Health Practice Project
FCSRT: Free and Cued Selective Remind Test
9-HPT: 9-Hole Peg Test
HVLT: Hopkins Verbal Learning Test Revised
MMSE: Mini Mental State Examination
MoCA: Montreal Cognitive Assessment
MS: Multiple sclerosis
PAL: Paired Associates Learning Task
PASAT: Paced Auditory Serial Addition Test
QbTest: Quantitative Behavioural Test
ROB: Risk of bias
RTI: Reaction time test
RVP: Rapid Visual Information Processing
SART: Sustained Attention to Response Task
SDMT: Symbol Digit Modalities Test
SWM: Spatial Working Memory Test
THC: Tetrahydrocannabinol
TMT: Trail Making Test
VLMT: Verbal Learning and Memory Test
VLT: Verbal Learning Test
WAIS: Wechsler Adult Intelligence Scale
